# Income inequality and life expectancy in Canada: New evidence from province-level panel regression, 1996–2019

**DOI:** 10.17269/s41997-025-01024-6

**Published:** 2025-05-21

**Authors:** Edgardo R. Sepulveda, Lindsay McLaren

**Affiliations:** 1 Toronto, ON Canada; 2 University of Calgary, Calgary, AB Canada

**Keywords:** Income inequality, Life expectancy, Social determinants of health, Political economy of health, Inégalité des revenus, Espérance de vie, Déterminants sociaux de la santé, Économie politique de la santé

## Abstract

**Objectives:**

Previous research on the association between income inequality and population health measures has yielded mixed results. This reflects, in part, the level of income inequality and surrounding political economic context of the setting in question. Previous research in Canada has not consistently identified an association between income inequality and population health measures. Those studies, however, largely focused on time periods prior to the manifestations of neoliberal policy reforms, which led to high levels of income inequality characterized by rising income at the top of the distribution. Our objective was to investigate the population-level association between income inequality and life expectancy in Canada during the years 1996–2019, a period of high after-tax income inequality in Canada.

**Methods:**

We used ordinary least squares panel multivariate regression analysis of publicly available aggregate data on after-tax income inequality and life expectancy for the 10 Canadian provinces during the period 1996–2019. We used an inequality variable that is sensitive to the disproportionate growth in income at the top of the income distribution (share of income held by top 5%); we took into account the proportion of the First Nations, Métis, and Inuit populations across provinces and over time; and we separately analyzed female, male, and total populations.

**Results:**

We found a robust, negative and statistically significant association where higher population-level after-tax income inequality was associated with lower average life expectancy in Canada.

**Conclusion:**

Our findings speak to the far-reaching consequences of neoliberalism, and to the need for public policy that will reduce income inequality in the interest of the public’s health.

## Résumé

**Objectifs:**

Les études passées sur l’association entre l’inégalité des revenus et les indicateurs de santé des populations ont donné des résultats mitigés. Cela reflète en partie le niveau d’inégalité des revenus et le contexte politico-économique du milieu en question. Les études passées menées au Canada ne font pas systématiquement état d’une association entre l’inégalité des revenus et les indicateurs de santé des populations. Ces études portent toutefois dans une large mesure sur des périodes antérieures aux manifestations des réformes stratégiques néolibérales qui ont entraîné des niveaux élevés d’inégalité des revenus, caractérisés par la croissance des revenus au haut de l’échelle. Nous nous sommes penchés sur l’association au niveau des populations entre l’inégalité des revenus et l’espérance de vie au Canada entre 1996 et 2019, une période d’inégalité élevée des revenus après impôt au pays.

**Méthode:**

Nous avons procédé par analyse de régression multivariée des moindres carrés ordinaires sur des données de panel agrégées, accessibles au public, sur l’inégalité des revenus après impôt et l’espérance de vie dans les dix provinces canadiennes pour la période de 1996 à 2019. Nous avons utilisé une variable d’inégalité sensible à la croissance disproportionnée des revenus au haut de l’échelle (la part des revenus détenue par les 5% supérieurs); nous avons tenu compte de la proportion des populations des Premières Nations, métisses et inuites par province et au fil du temps; et nous avons analysé séparément les populations féminines, masculines et totales.

**Résultats:**

Nous avons trouvé une association négative robuste significative selon laquelle l’inégalité élevée des revenus après impôt au niveau des populations était associée à une espérance de vie moyenne plus faible au Canada.

**Conclusion:**

Nos constats témoignent des conséquences profondes du néolibéralisme et de la nécessité d’une politique publique qui réduise l’inégalité des revenus dans l’intérêt de la santé publique.

## Introduction

Publication of the books *The Spirit Level* (Wilkinson & Pickett, 2010) and *Capital in the Twenty-First Century* (Piketty, 2014) signalled academic and popular interest in income inequality, including its implications for the public’s health. Situated within scholarship on the social determinants and political economy of health (Chernomas & Hudson, 2013; Raphael et al., 2020), this work connects population health outcomes with upstream social, economic, and political systems and structures, including dynamics of power, which shape inequality and population well-being through public policy decisions.

Many studies have examined whether population-level income inequality is associated with population health measures. Based on a large meta-analysis of studies published up to 2008, Kondo et al. (2009) identified a modest adverse effect of income inequality (measured by the Gini coefficient—an index that measures inequality and varies between 0 [perfect equality] and 1 [when one person has 100% of the income]) on mortality and self-rated health. Statistical associations were stronger among studies conducted using data after 1990; they were also stronger among studies where the Gini coefficient was higher, leading the authors to suggest a threshold effect where adverse impacts on population health emerge at a certain level of inequality. A 2015 narrative review by Pickett and Wilkinson (2015) likewise concluded that more recent studies provide evidence that population health tends to be worse in more unequal societies; these authors argued that the association is consistent with causal criteria. On the other hand, a 2024 systematic review and meta-analysis identified a small association between income inequality and poor self-rated health and all-cause mortality and argued that the association is not consistent with causality, although they noted that the available evidence remains methodologically limited (Shimonovich et al., 2024). Recent primary research has supported an association between higher income inequality (Gini) and higher (worse) COVID-19 mortality across OECD countries (Sepulveda & Brooker, 2021).

Canadian studies to date have not shown a robust association between income inequality and population health measures. In a comparison of Canada and the United States (USA) using Canadian data from 1990 to 1992, Ross et al. (2000) found, in contrast to the USA, no significant association among Canadian provinces and metropolitan areas between income inequality (defined as % income held by bottom 50% of households) and mortality, which they attributed to lower income inequality in Canada at the time as well as a better system of distribution of social and economic resources. A Canadian time series analysis by Laporte and Ferguson (2003) likewise found no statistically significant association between income inequality (Gini) and age-adjusted mortality, based on a province-level panel regression of data from 1980 to 1997. A study by Auger et al. (2012) hypothesized that the apparent lack of effect could reflect the inclusion of immigrants who tend to be healthier and mask an overall effect, and indeed they found that when they stratified by immigrant status an effect was apparent among non-immigrants only based on data from 1991 to 2001. Finally, a Canadian study by Liu and Dutton (2021), although focused primarily on government spending, also did not show an adverse unadjusted effect of income inequality, based on the Gini coefficient, on five population health outcomes in a province-level analysis of data from 1981 to 2017, for males or females.

As argued by Coburn (2004) among others, income inequality must be situated within the broader political economy context, including the spread of neoliberalism and corresponding changes to the relationships between class structure, economies, and human well-being. Neoliberal capitalism became the dominant political economic paradigm in much of the Global North in the early 1980s, although its ideological roots go back much further (Harvey, 2005). The class dominance of business brought on by economic globalization underpinned economic and social policy that collectively served to weaken labour power, exacerbate social and economic inequality, and undermine the public and social institutions that could mitigate the effects of poverty and inequality (Coburn, 2004; Osberg, 2020).

Compared to other settings such as the USA and the United Kingdom, neoliberalism emerged later in Canada (Himelfarb, 2024). This, Osberg (2020) notes, was a function of Canadian historical circumstances. Canada’s Keynesian economic period (approximately 1946–1981) was characterized by an emphasis on full employment and a commitment to the view that sharing the benefits of economic growth was an essential function of government. That commitment was embodied in Canada’s tax/transfer system. The result was that, while inequality based on market income in Canada began to rise with the emergence of global neoliberalism in the early 1980s, tax/transfer policies developed during the Keynesian period initially served to offset rising market inequality, and inequality based on after-tax income did not begin to increase until the mid-1990s (Osberg, 2020).

Federal Progressive Conservative Prime Minister Brian Mulroney, elected in 1984, initiated large-scale deregulation, major tax reforms, deep cuts to spending on social programs, and consequential trade negotiations with the USA (Himelfarb, 2024). However, Himelfarb (2024) argues that while Mulroney’s government “set the course” for neoliberalism, it was the Liberal governments of the 1990s who gave neoliberalism in Canada “a big push forward”, perhaps most notably through the Chrétien government’s 1995 federal budget that embodied a significant shift towards a government whose primary role was to facilitate business and reduce the debt, with austerity put forth as the only viable solution. Collectively, economic and social policy during this period served to “severely weaken Canada’s major redistributive programs” (Himelfarb, 2024, p. 220), including the tax/transfer system, which resulted in an increase in after-tax income inequality (Osberg, 2020).

Our study objective was thus to investigate the population-level association between after-tax income inequality and life expectancy in Canada in this neoliberal context. Our study updates and expands on previous Canadian studies that have not found a robust relationship between income inequality and population health measures. First, it covers the years 1996–2019, thus including a period during which the manifestations of neoliberal economic social and economic policy in Canada are evident. Second, it uses an inequality variable that is sensitive to the disproportionate increases at the top of the income distribution which characterize the rising inequality during this period. Third, in recognition of life expectancy differences between Indigenous and non-Indigenous populations (Tjepkema et al., 2019), which reflect the ongoing harms of colonial systems and structures, our analysis includes the province-level proportion of First Nations, Métis, and Inuit populations. These Indigenous populations of Canada have unique health profiles and strengths, which have been damaged by colonialism and racism; meaningful engagement with Indigenous data and communities is one small aspect of Truth and Reconciliation. Our analysis also includes median income and (in sensitivity analyses) poverty levels, as covariates. Finally, this study builds on previous work by including sex-stratified analyses and by employing two-way province and time fixed effects and autocorrelation-corrected standard errors to yield robust estimates.

## Methods

We used ordinary least squares (OLS) panel multivariate regression to analyze the association between after-tax income inequality and life expectancy during the 1996–2019 period for the female, male, and total population across the 10 Canadian provinces (i.e., Alberta, British Columbia, Manitoba, New Brunswick, Newfoundland and Labrador, Nova Scotia, Ontario, Prince Edward Island, Quebec, and Saskatchewan). We use terms “female” and “male” to respect that the variable available presents sex, rather than gender. Territorial and sub-provincial (e.g., municipal) data were not available for our variables over the study period. We selected 1996 as the beginning of our study period to permit updating earlier studies and to capture a period where, as described above, manifestations of neoliberalism, including rising inequality of after-tax income, were evident in Canada. We selected 2019 as the end year to restrict our analysis to the pre-COVID-19 period.

### Study variables and data sources

**Fig. 1:**
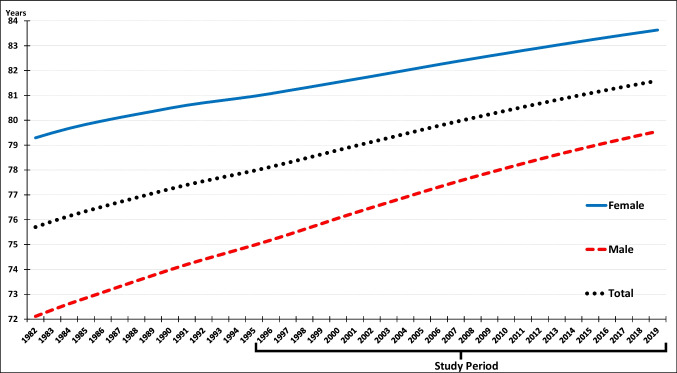
Female, male, and total life expectancy (10-province averages of LOESS-smoothed 1982–2019 data)

Life expectancy is the outcome variable. Annual life expectancy data for the female, male, and total population was sourced from the Canadian Human Mortality Database (Department of Demography, Université de Montréal, 2025). Figure 1 shows the 10-province averages for life expectancy for extended (1982–2019) and study (1996–2019) periods. As shown, the average life expectancy across the 10 provinces increased during the study period for the female, male, and total populations, but at different rates. To account for these differential trends, we ran separate regression models for female, male, and total populations.

**Fig. 2:**
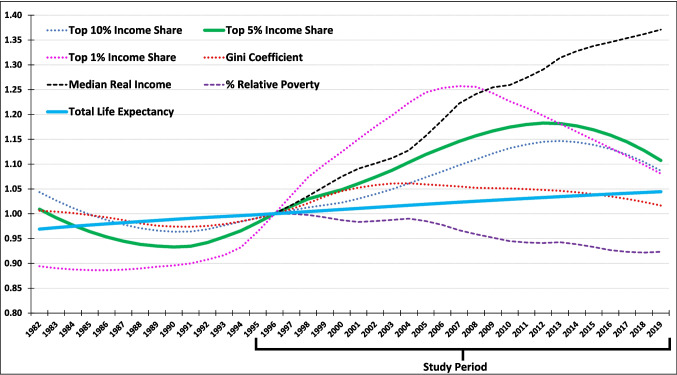
Indices of total life expectancy; income; shares of top 10%, 5%, and 1%; Gini coefficient; and relative poverty (10-province averages of LOESS-smoothed 1982–2019 data; indexed to 1996 [1996 = 1.00]). Income and income inequality variables are based on after-tax income

Income inequality is the primary predictor variable. We focused on after-tax income inequality because (a) it reflects the income that people have in their pockets and is thus more meaningful, and (b) the after-tax income distribution reflects the flattening that occurs through the tax/transfer system and thus avoids inflated and possibly misleading estimates of market income inequality. Increases in income inequality during most of the study period primarily reflect income gains among those at the top of the income distribution (Osberg, 2020). Accordingly, we sought a measure that was sensitive to this pattern. We used the share of total income held by the top 5% of the income distribution (Statistics Canada, 2022a). Figure 2 shows the averages across the 10 provinces for inequality based on the top 1%, top 5%, and top 10% shares for the extended period 1982–2019, along with the Gini coefficient (Statistics Canada, 2023a).

As shown, after-tax income inequality based on all measures decreased until the early 1990s, after which they all began to increase but in different ways. Income inequality based on the top 1%, 5%, and 10% shares increased to a much greater extent than for the Gini coefficient, which is less sensitive to changes at the top or bottom of the income distribution. Income inequality based on the top 1% increased sooner and to a greater extent than inequality based on the top 5% and 10%; it also began to decrease earlier, around the 2008 global financial crisis. The proportion of income that takes the form of capital (i.e., dividends and profits from assets such as real estate or stocks) rather than labour (i.e., income from a wage or salary) tends to increase further up the income distribution, so incomes at the very top of the distribution rise and fall with the business cycle and are thus more volatile, although this is substantially muted for after-tax income (Osberg, 2020). Provincial-level data availability is another consideration, ranging from complete coverage during our study period for the top 10% and top 5%, to consistent coverage of only the most populous province, Ontario, for the top 0.01%. Data for the top 1% for the least populous province, Prince Edward Island, was not available for the years 1996–1998. Because of the top 1%’s relatively greater volatility, coupled with missing data for the top 1%, we opted to use income inequality for the top 5% as the inequality variable for our main models, and the top 1% (for which we intrapolated the missing data for PEI to have a balanced sample), top 10%, and the Gini coefficient as sensitivity analysis. For context, Fig. 2 also shows the share of the population with income below 50% of the total population median after-tax income (an estimate of % relative poverty), based on annual income estimates for census families and individuals (Statistics Canada, 2023b), the median real after-tax income (see below) and the average total (female and male) life expectancy. All variables in Fig. 2 are indexed to 1996, the first year of our study period.

**Fig. 3:**
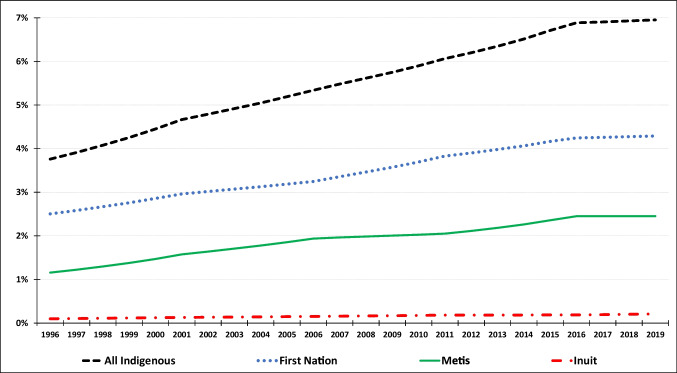
Indigenous populations (% of total population) (10-province averages of 1996–2019 data)

With the objective of robustly estimating the independent association between income inequality and life expectancy, in our main models, we included two additional variables (covariates) that may be expected to affect average life expectancy. First is the median after-tax income (Statistics Canada, 2023c) in 2019 constant dollars. Second is the proportion of Indigenous population for each province, based on overall counts, for total Indigenous population (model B), and for First Nations, Métis, and Inuit populations separately (model C), presented in Fig. 3. The only available data source designed to cover all Indigenous peoples at this level of disaggregation across provinces over time is the 5-year census, based on which we compiled Indigenous populations for 1996, 2001, 2006, 2011, 2016, and 2021. We estimated inter-census populations by calculating the respective 5-year cumulative annual growth rate and applying it to each of the inter-census years. For the denominator in both models, we used the annual provincial population estimates (Statistics Canada, 2022b). Figure 3 highlights two issues that we develop in the Robustness and sensitivity checks section: that the Métis and First Nations populations have grown faster than the general population, and that the Inuit population is relatively very small.

### Statistical analysis

Our OLS panel multivariate regression analysis takes advantage of variations over time in provincial after-tax income inequality and provincial life expectancy, to assess whether higher after-tax income inequality is, on average, associated with lower life expectancy across Canadian provinces, after accounting for variations in provincial income and the proportion of Indigenous provincial population. We included province fixed effects to account for unobserved factors that differed between provinces but were constant over time. Because all variables change over time, we included time fixed effects to account for unobserved factors shared by all provinces at a specific point in time. Given the relatively limited number of observations in our data set (240 province-years), we selected 4-year interval time increments (dummy variables) to maintain degrees of freedom (Torre & Myrskylä, 2014) (i.e., 1996–1999, 2001–2004). Fixed effects are designed to control for time and province-specific factors that may confound the relationship between life expectancy and inequality and provide biased results. We provide the results of not employing this two-way province and time fixed effects in the Robustness and sensitivity checks section. Finally, to correct for autocorrelation, we report “Driscoll-Kraay” standard errors.

Before the regression analysis, we took the natural logarithm of the life expectancy and median income variables to allow for estimation of elasticities and to control for heteroscedasticity. For the top 5% income share and population proportion variables, we multiplied them by 10 to ease the interpretation of coefficient results. To reduce undue influence of year-over-year variability, especially present in relatively small-population provinces, we applied locally estimated scatterplot smoothing (LOESS) (we provide actual [unsmoothed] data in the Appendix for the smoothed data in Figs. 1 and 2).

## Results

Table 1 provides descriptive statistics for all study variables. The statistical association between after-tax income inequality and life expectancy based on three regression models is presented in Table 2. These models are also designed to assess the robustness of the coefficient results across several specifications: model A includes after-tax income inequality and median real after-tax income only; model B adds the proportion of total Indigenous population; and model C adds the proportion of First Nations, Métis, and Inuit populations separately. Results for each model are provided for the female, male, and total populations, for a total of nine regressions.

**Table 1:** Descriptive statistics for all study variables (LOESS-smoothed). Income and income inequality variables are based on after-tax income

*N* = 240	Units	Mean	Std. dev	Min	Max	Norm. SD
Female life expectancy	Years	82.39	1.12	79.97	84.90	0.014
Male life expectancy	Years	77.54	1.63	74.48	81.43	0.021
Total life expectancy	Years	79.98	1.37	77.19	83.17	0.017
Top 10% income share	% provincial income	0.253	0.085	0.145	0.480	0.336
Top 5% income share	% provincial income	0.161	0.066	0.085	0.351	0.408
Top 1% income share	% provincial income	0.062	0.034	0.023	0.179	0.551
Gini coefficient	% provincial income	0.297	0.016	0.242	0.331	0.054
% relative poverty	% provincial income	0.180	0.022	0.130	0.228	0.124
Median real income	Constant 2019 dollars	48,418	6,478	35,837	67,312	0.134
% all Indigenous	% provincial population	0.0553	0.0481	0.0067	0.1700	0.869
% First Nations	% provincial population	0.0346	0.0299	0.0058	0.1032	0.863
% Métis	% provincial population	0.0191	0.0189	0.0008	0.0699	0.988
% Inuit	% provincial population	0.0016	0.0033	0.0001	0.0142	2.122

**Table 2:** Results of ordinary least squares (OLS) regression, with life expectancy regressed on income inequality (top 5% income share), median real income, and proportion Indigenous population, in 10 Canadian provinces (1996–2019). Income and income inequality variables are based on after-tax income

	Model A			Model B			Model C		
Life expectancy (by sex, years)	Female	Male	Total	Female	Male	Total	Female	Male	Total
Regression #	#1	#2	#3	#4	#5	#6	#7	#8	#9
Variables									
Top 5% income share	− 0.0093*	− 0.0138**	− 0.0113**	− 0.0089**	− 0.0133*	− 0.0109*	− 0.0089*	− 0.0149**	− 0.0117**
	(0.0033)	(0.0045)	(0.0037)	(0.0030)	(0.0050)	(0.0039)	(0.0033)	(0.0048)	(0.0038)
Median real income	0.0070	0.0321	0.0182	0.0263	0.0549*	0.0386 +	0.0234	0.0524 +	0.0354
	(0.0200)	(0.0290)	(0.0244)	(0.0157)	(0.0255)	(0.0211)	(0.0163)	(0.0266)	(0.0217)
% all Indigenous				− 0.0262***	− 0.0311**	− 0.0277***			
				(0.0033)	(0.0088)	(0.0058)			
% First Nations							− 0.0352**	− 0.0252*	− 0.0307**
							(0.0097)	(0.0100)	(0.0102)
% Métis							− 0.0285*	− 0.0570***	− 0.0424***
							(0.0121)	(0.0091)	(0.0081)
% Inuit							0.1323	0.0625	0.1243
							(0.0832)	(0.1036)	(0.0977)
Fixed effects									
Province	Yes	Yes	Yes	Yes	Yes	Yes	Yes	Yes	Yes
4-year interval	Yes	Yes	Yes	Yes	Yes	Yes	Yes	Yes	Yes
Fit statistics									
Adjusted *R*^2^	0.929	0.930	0.937	0.943	0.938	0.946	0.945	0.940	0.949
Observations	240	240	240	240	240	240	240	240	240

Autocorrelation-corrected “Driscoll-Kraay” (*L* = 2) standard errors in parentheses. Non-binary variables (life expectancy and median real income) in logarithmic form (ln), binary variables (top 5% income share and population proportions) multiplied by 10. Significance codes: *** = 0.001, ** = 0.010, * = 0.050, ^+^ = 0.100

Model A, containing only income inequality (top 5% share) and median real income, shows a negative and statistically significant association between after-tax income inequality and average life expectancy for the female, male, and total populations (i.e., higher after-tax income inequality, lower average life expectancy).

Models B and C add the proportion of Indigenous population, and the proportion of First Nations, Métis, and Inuit populations, respectively. The negative and significant income inequality (top 5% share) coefficients seen in model A persist in models B and C, indicating they are robust to the addition of the Indigenous population covariates. The Indigenous population coefficients in model B are negative and statistically significant (i.e., higher proportion Indigenous population, lower life expectancy). In model C, the coefficients for the First Nations and Métis populations are all negative and statistically significant. In contrast, the Inuit population coefficient is not statistically significant, which likely reflects the relatively very small Inuit population, as discussed earlier, and further modelled below.

### Robustness and sensitivity checks

We undertook a series of robustness and sensitivity checks (see the Appendix). First, for the after-tax inequality variable, we re-ran all regressions using the top 10% and the top 1% income shares and the Gini coefficient. The results for the top 10% income share (Appendix model D) were consistent with our main results in Table 2, with inequality being negatively and statistically significantly associated with life expectancy. The top 1% income share coefficient (Appendix model E) was negative but not statistically significant. This is not unexpected considering the greater volatility of the top 1% variable. In contrast, results using the Gini coefficient (Appendix model F) were a mix of mostly non-significant positive and negative effects, which may reflect its different evolution over the study period.

Second, in response to a common objection that the income inequality–population health relationship is an artefact of poverty (Lynch et al., 2004; Pickett & Wilkinson, 2015), we re-ran all regressions adding the % relative poverty variable (Appendix model G). The results were consistent with our main results in Table 2; that is, the association between higher after-tax income inequality and lower life expectancy persisted, with % relative poverty in the model, for all nine regressions.

Third, we re-ran the main models without employing province and time fixed effects (Appendix model H). The results are consistent with the main models in Table 2 and show higher levels of statistical significance, mostly driven by an increase in the size of the coefficients. The use of two-way fixed effects, therefore, confirms the robustness of the estimates.

Fourth, we ran several robustness and sensitivity checks that concerned the Indigenous population variables. There are well-known data quality limitations associated with Indigenous population census estimates, which themselves reflect colonial practices that have harmed relationships with Indigenous communities. These include inconsistent coverage due to some geographic areas not being enumerated, as well as changing wording and format of self-reported Indigenous identity questions (Feir & Hancock, 2016). For the Indigenous population variables, we re-ran regressions that (1) accounted for response mobility; (2) disaggregated Indigenous population based on self-reported Indian Status; and (3) excluded the Inuit population as a separate variable.

#### Response mobility:

**Fig. 6:**
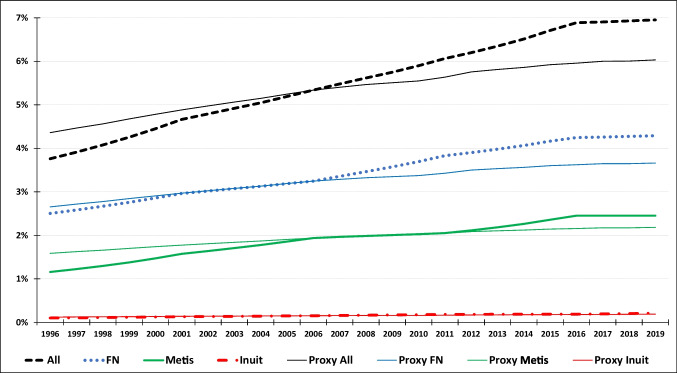
Indigenous populations (% of total population) census-based and proxy populations (10-province averages of 1996–2019 data)

Response mobility refers to the proportion of the population that identifies as Indigenous varying over time, such that observed increases in the Indigenous population highlighted in Fig. 6 reflect both response mobility and demographic factors. For example, O’Donnell and LaPointe (2019) show that much of the increase in population from one period to the next is due to a net inflow of persons who did not identify as Indigenous in the first period but did so in the second period (the incoming group), with the result that the response mobility phenomenon tended to increase the Indigenous population faster than demographic factors (e.g., birth rates). Time series analysis of population health should account for the issue of response mobility (Health Canada, 2012), including because the socio-economic characteristics of the incoming group are different from those who have consistently reported Indigenous identity (Siggner, 2003). As such, life expectancy of the incoming group is likely to be different as well. However, the most extensive study to calculate the degree of response mobility only covers 10 years (O’Donnell & LaPointe, 2019). With a view to assessing the sensitivity of our results, we constructed three *proxy* Indigenous population series designed to minimize the response mobility phenomenon. For the First Nations, Métis, and Inuit groups, we set the 2006 populations equal to the 2006 census data and calculated other years based on the province and year-specific annual population change in the Indian Status population (Indigenous Services Canada, 2022a); that is, the legal standing of a person who is registered in the Indian Register pursuant to the Indian Act (Indigenous Services Canada, 2022b). Figure 6 in the Appendix presents the proxy as well as the census-based Indigenous population estimates. The results of using these proxy population variables (Appendix model I) were generally consistent with those in Table 2, including a negative and significant association between income inequality and life expectancy. The sign and significance for the Métis population variable changed compared to the results in Table 2, which may reflect the relatively larger response mobility for this population group.

#### Disaggregation of Indigenous population:

There are several different ways of disaggregating the Indigenous population in addition to First Nations, Métis, and Inuit. One is by self-reported Indian Status (status, non-status). The results of this regression (Appendix model J) were generally consistent with those in Table 2, including of a negative and significant association between income inequality and life expectancy.

#### Exclusion of Inuit population:

We re-ran the regressions excluding the Inuit population variable due to its relatively very small size (Appendix model K), and the results were generally consistent with those in Table 2.

## Discussion

We identified a robust, negative, and statistically significant association between after-tax income inequality and average life expectancy in Canada, over an extended period (1996–2019) during which income inequality in Canada, due to the manifestation of neoliberal ideology and policy, was relatively high (Himelfarb, 2024; Osberg, 2020).

This is the first Canadian study to identify such an effect. Earlier Canadian studies, using cross-sectional or panel analysis, included study periods during which income inequality, regardless of how it was measured, was relatively lower. This speaks to the importance of updating research to reflect changing political economy contexts and priorities, including the neoliberal social and economic reforms that took hold globally in the early 1980s and whose effects—including rising after-tax income inequality—began to manifest in Canada in the mid-1990s. More recent studies of income inequality and population health in Canada have often used the Gini coefficient. While this is a robust and familiar measure of a population’s income distribution, it has not captured the recent growth in income inequality, which is known to reflect disproportionate increases among those at the top of the income distribution (Osberg, 2020).

It is well established that dominant approaches to social and economic policy under neoliberalism, including austerity and privatization of public services, and deregulation of industry and labour markets, have significantly eroded social and ecological determinants of health (Ruckert & Labonté, 2014; Schrecker & Bambra, 2015). Our findings suggest that the recent high levels of after-tax income inequality have reached a threshold level above which it has a negative effect on population health, as hypothesized by Kondo et al. (2009) when levels of inequality in many places were lower. We echo recent work arguing that meaningful engagement with political economy should be a priority for those concerned with the public’s health in Canada (McLaren & Mykhalovskiy, 2024).

Another way in which our study builds on previous work is our inclusion of the Indigenous population proportion in each province. We found that the negative statistical association between after-tax income inequality and life expectancy persisted in models that included the proportion of all Indigenous populations as well as in models where the First Nations, Métis, and Inuit populations were considered separately. While this disaggregation of Indigenous populations builds on previous work, we recognize that it ignores important cultural and linguistic diversity among the communities within each group (TRC, 2015) as well as other dimensions of diversity such as sex and gender, which intersect in complex ways to affect life expectancy.

Those models also demonstrated that a higher proportion of Indigenous population was associated with lower average life expectancy, consistent with previous work that found shorter life expectancies for Indigenous peoples (Tjepkema et al., 2019). Colonial and racist structures and systems are the fundamental causes of these shorter life expectancies, and those structures are embodied in the data we used here—they underpin, for example, what might be seen as “data limitations” due to inconsistent coverage, which in fact reflect harmful colonial relationships. While data harmonization and sharing to permit comparisons with non-Indigenous populations is desirable, it is imperative that this occurs within Indigenous-led data gathering processes, for which powerful frameworks and platforms exist including OCAP (ownership, control, access, and possession) principles, put forth by the First Nations Information Governance Centre (FNIGC, 2022). We support Feir and Hancock’s (2016) plea for quantitative social scientists, including those in public health, in Canada and beyond, to “Answer the Call” to further the project of Truth and Reconciliation by working with and meaningfully integrating data related to Indigenous people in their research.

## Contributions to knowledge

What does this study add to existing knowledge?

- We found a robust, negative, statistically significant association between after-tax income inequality and life expectancy in Canada (higher inequality, lower life expectancy).
- This association has not previously been demonstrated in Canada. This is because earlier studies largely focused on time periods prior to the manifestations of neoliberal social and economic reforms, which led to, among other things, high levels of income inequality characterized by rising income at the top of the income distribution.

What are the key implications for public health interventions, practice, or policy?

- Our findings provide an evidentiary foundation for public policy to reduce income inequality in the interest of the public’s health.
- When thinking about policy for health, our findings underscore that it is important for those concerned with the public’s health in Canada to engage meaningfully and critically with political economy.

## Figures and Tables

**Fig. 4:**
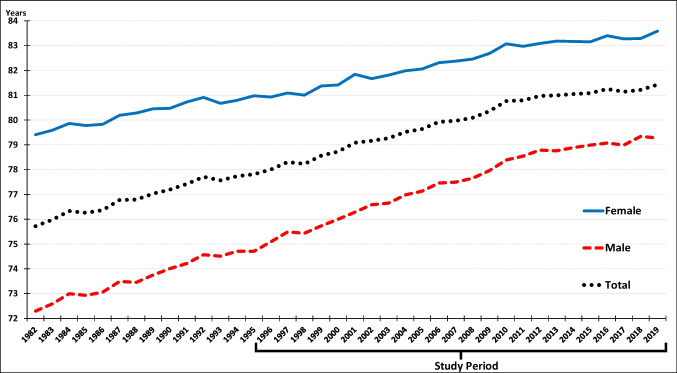
Female, male, and total life expectancy (10-province averages 1982–2019 data)

**Fig. 5:**
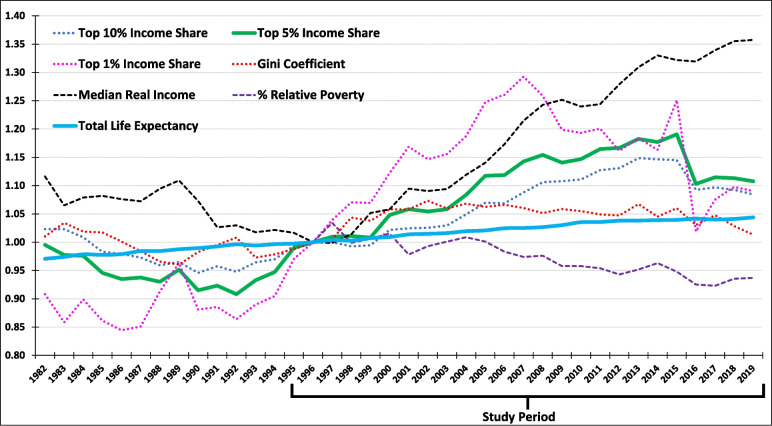
Indices of total life expectancy; income; shares of top 10%, 5%, and 1%; Gini coefficient; and relative poverty (10-province averages; indexed to 1996 [1996 = 1.00]). Income and income inequality variables are based on after-tax income

## Data Availability

Data are available from Statistics Canada and other publicly available sources, as indicated in the manuscript.

## Appendix

This section presents a selection of the regressions and charts described in the Robustness and sensitivity checks section.

Model D includes the top 10% income share as the inequality variable, instead of the top 5% income share included in Table 2. Model E includes the top 1% income share as the inequality variable and model F includes the Gini coefficient as the inequality variable. Income and income inequality variables are based on after-tax income.

**Table:**

	Model D			Model E			Model F		
Life expectancy (by sex, years)	Female	Male	Total	Female	Male	Total	Female	Male	Total
Regression #	#10	#11	#12	#13	#14	#15	#16	#17	#18
Variables									
Top 10% income share	− 0.0104***	− 0.0156**	− 0.0128**						
	(0.0024)	(0.0046)	(0.0034)						
Top 1% income share				− 0.0037	− 0.0055	− 0.0043			
				(0.0039)	(0.0058)	(0.0047)			
Gini coefficient							0.0021	0.0074	0.0059
							(0.0047)	(0.0075)	(0.0062)
Median real income	0.0443**	0.0823**	0.0609*	− 0.0034	0.0104	0.0018	− 0.0091	0.0012	− 0.0055
	(0.0149)	(0.0255)	(0.0219)	(0.0126)	(0.0189)	(0.0155)	(0.0100)	(0.0159)	(0.0122)
% all Indigenous	− 0.0234***	− 0.0269**	− 0.0243***	− 0.0268***	− 0.0320***	− 0.0284***	− 0.0272***	− 0.0340**	− 0.0301***
	(0.0039)	(0.0091)	(0.0063)	(0.0032)	(0.0077)	(0.0049)	(0.0043)	(0.0093)	(0.0066)
Fixed effects									
Province	Yes	Yes	Yes	Yes	Yes	Yes	Yes	Yes	Yes
4-year interval	Yes	Yes	Yes	Yes	Yes	Yes	Yes	Yes	Yes
Fit statistics									
Adjusted *R*^2^	0.946	0.942	0.950	0.939	0.934	0.942	0.938	0.934	0.942
Observations	240	240	240	240	240	240	240	240	240

Autocorrelation-corrected “Driscoll-Kraay” (*L* = 2) standard errors in parentheses. Non-binary variables (life expectancy and median real income) in logarithmic form (ln), binary variables (top 10% income share, top 1% income share, Gini coefficient, and population proportions) multiplied by 10. Significance codes: *** = 0.001, ** = 0.010, * = 0.050, ^+^ = 0.100

Model G adds the % relative poverty variable to the main model in Table 2. Model H uses the same variables as model B in Table 2, but does not include either the province or time fixed effects. Income, relative poverty, and income inequality variables are based on after-tax income.

**Table:**

	Model G			Model H		
Life expectancy (by sex, years)	Female	Male	Total	Female	Male	Total
Regression #	#19	#20	#21	#22	#23	#24
Variables						
Top 5% income share	− 0.0080**	− 0.0110*	− 0.0095**	− 0.0536***	− 0.0485***	− 0.0512***
	(0.0024)	(0.0041)	(0.0030)	(0.0025)	(0.0021)	(0.0022)
% relative poverty	0.0006	0.0017*	0.0010 +			
	(0.0005)	(0.0006)	(0.0006)			
Median real income	0.0379 +	0.0859*	0.0572 +	0.4189***	0.4124***	0.4157***
	(0.0207)	(0.0326)	(0.0286)	(0.0010)	(0.0006)	(0.0008)
% all Indigenous	− 0.0251***	− 0.0283**	− 0.0261***	− 0.0316***	− 0.0302***	− 0.0316***
	(0.0037)	(0.0093)	(0.0062)	(0.0046)	(0.0040)	(0.0043)
Fixed effects						
Province	Yes	Yes	Yes	No	No	No
4-year interval	Yes	Yes	Yes	No	No	No
Fit statistics						
Adjusted *R*^2^	0.944	0.941	0.948	− 2.538	0.023	− 0.804
Observations	240	240	240	240	240	240

Autocorrelation-corrected “Driscoll-Kraay” (*L* = 2) standard errors in parentheses. Non-binary variables (life expectancy and median real income) in logarithmic form (ln), binary variables (top 5% income share, % relative poverty, and population proportions) multiplied by 10. Significance codes: *** = 0.001, ** = 0.010, * = 0.050, ^+^ = 0.100

Model I includes the Proxy First Nations, Métis, and Inuit population variables (as described in the Robustness and sensitivity checks section), instead of the census-based First Nations, Métis, and Inuit population variables included in Table 2. Model J disaggregates the Indigenous population based on self-reported Indian Status. Model K excludes the Inuit population variable as a separate variable. Income and income inequality variables are based on after-tax income.

**Table:**

	Model I			Model J			Model K		
Life expectancy (by sex, years)	Female	Male	Total	Female	Male	Total	Female	Male	Total
Regression #	#25	#26	#27	#28	#29	#30	#31	#32	#33
Variables									
Top 5% income share	− 0.0107**	− 0.0152**	− 0.0130**	− 0.0088**	− 0.0142**	− 0.0114**	− 0.0096**	− 0.0152**	− 0.0124**
	(0.0029)	(0.0044)	(0.0035)	(0.0028)	(0.0047)	(0.0036)	(0.0032)	(0.0047)	(0.0038)
Median real income	0.0430**	0.0656*	0.0538*	0.0262	0.0556*	0.0389 +	0.0258	0.0535 +	0.0376 +
	(0.0151)	(0.0252)	(0.0193)	(0.0155)	(0.0256)	(0.0211)	(0.0162)	(0.0262)	(0.0216)
% First Nations							− 0.0232***	− 0.0195 +	− 0.0194*
							(0.0052)	(0.0105)	(0.0076)
% Métis							− 0.0380**	− 0.0615***	− 0.0512***
							(0.0112)	(0.0103)	(0.0094)
% Inuit									
% status				− 0.0272 +	− 0.0193	− 0.0219 +			
				(0.0104)	(0.0153)	(0.0127)			
% non-status				− 0.0251	− 0.0449*	− 0.0346*			
				(0.0155)	(0.0174)	(0.0154)			
% proxy First Nations	− 0.1244***	− 0.0228	− 0.0841***						
	(0.0139)	(0.0361)	(0.0100)						
% proxy Métis	0.1045***	− 0.0724	0.0289						
	(0.0149)	(0.0519)	(0.0196)						
% proxy Inuit	− 0.1801***	− 0.0725	− 0.1089 +						
	(0.0359)	(0.1112)	(0.0633)						
Fixed effects									
Province	Yes	Yes	Yes	Yes	Yes	Yes	Yes	Yes	Yes
4-year interval	Yes	Yes	Yes	Yes	Yes	Yes	Yes	Yes	Yes
Fit statistics									
Adjusted *R*^2^	0.950	0.941	0.952	0.943	0.938	0.946	0.944	0.940	0.948
Observations	240	240	240	240	240	240	240	240	240

Autocorrelation-corrected “Driscoll-Kraay” (*L* = 2) standard errors in parentheses. Non-binary variables (life expectancy and median real income) in logarithmic form (ln), binary variables (top 5% income share and population proportions) multiplied by 10. Significance codes: *** = 0.001, ** = 0.010, * = 0.050, ^+^ = 0.100

Figure 4 presents the raw (unsmoothed) series of female, male, and total life expectancy (10-province averages 1982–2019), and as such complements Fig. 1, which presents the smoothed series.

Figure 5 presents the raw (unsmoothed) series of indices of total life expectancy; income; shares of top 10%, 5%, and 1%; Gini coefficient; and relative poverty, and as such complements Fig. 2, which presents the smoothed series.

Figure 6 presents the proxy Indigenous populations used for sensitivity analyses, as well as the census-based Indigenous populations presented in Fig. 3.

## Code availability

Available from the corresponding author upon reasonable request.
